# Retraction: Tongyi Lu, Binhua Wu, Yunfei Yu, Wenhui Zhu, Simin Zhang, Yinmei Zhang, Jiaying Guo, Ning Deng. Blockade of ONECUT2 expression in ovarian cancer inhibited tumor cell proliferation, migration, invasion and angiogenesis. *Cancer Sci*. 2018; 109: 2221–2234. https://doi.org/10.1111/cas.13633

**DOI:** 10.1111/cas.16108

**Published:** 2024-02-11

## Abstract

The above article published online on 08 May 2018 in Wiley Online Library (wileyonlinelibrary.com), has been retracted by agreement between the journal Editor‐in‐Chief, Masanori Hatakeyama, the Japanese Cancer Association and John Wiley and Sons Ltd. The retraction has been agreed upon following an investigation into concerns raised by a third party, which identified multiple duplications and splicing in Figures 3, 5 and 6 in the article. Although the authors corrected some of these images after repeating the experiments (Correction: https://doi.org/10.1111/cas.15012), they failed to provide an unedited source image for Figure 6 upon request or a satisfactory explanation. Given the number and extent of the identified issues, the editors have lost confidence in the data and have therefore decided to retract it. The authors were informed of the decision to retract but remained unresponsive.

The above article published online on 08 May 2018 in Wiley Online Library (wileyonlinelibrary.com), has been retracted by agreement between the journal Editor‐in‐Chief, Masanori Hatakeyama, the Japanese Cancer Association and John Wiley and Sons Ltd. The retraction has been agreed upon following an investigation into concerns raised by a third party, which identified multiple duplications and splicing in Figures 3, 5 and 6 in the article. Although the authors corrected some of these images after repeating the experiments (Correction: https://doi.org/10.1111/cas.15012), they failed to provide an unedited source image for Figure 6 upon request or a satisfactory explanation. Given the number and extent of the identified issues, the editors have lost confidence in the data and have therefore decided to retract it. The authors were informed of the decision to retract but remained unresponsive.

